# Cognitive Processes of ESL Learners in Pragmatic Role-Play Tasks in Academic Settings

**DOI:** 10.3389/fpsyg.2021.586588

**Published:** 2021-08-30

**Authors:** Nick Zhiwei Bi

**Affiliations:** College of Foreign Languages, University of Shanghai for Science and Technology, Shanghai, China

**Keywords:** cognitive processes, metacognitive and cognitive strategies, pragmatic strategies, L2 pragmatic competence, task-based language teaching, role-plays

## Abstract

The purpose of the study was to investigate the cognitive processes of English as second language (L2) learners that are involved in their task-based pragmatic performances in academic settings. This study, therefore, examined the cognitive processes of 30 English L2 learners when engaging in various role-play-based pragmatic performances, such as requesting a recommendation letter from a professor and negotiating an agreeable meeting time with classmates. The qualitative analyses of the retrospective verbal reports (RVRs) data of the participants indicated that the learners employed a series of cognitive, metacognitive, and pragmatic strategies when accomplishing various speech acts (e.g., requests and refusals). This study hoped to make two new contributions to the field. First, the study provided empirical evidence to validate the theoretical taxonomy of the strategy use of learners in L2 pragmatics. Additionally, the theoretical foundations of current research on cognitive processes are primarily informed by pragmatic theories. Thus, the study aims to explicate a more comprehensive view of the cognitive processes of L2 learners in pragmatic performances by employing the theories from both pragmatic and learner strategy perspectives.

## Introduction

Various communicative language ability models (see Canale and Swain, 1980; Bachman and Palmer, 1996, 2010) have suggested that language ability includes both language knowledge and strategic competence (e.g., the metacognitive and cognitive strategies of L2 learners). These strategies are “conscious or semi-conscious thoughts and actions deployed by learners, often with the intention of enhancing their knowledge of, and facility with an L2” (Ishihara and Cohen, 2010, p. 228). Pragmatic competence has been widely recognized as an essential ability to use language appropriately in a social context (Taguchi and Roever, 2017). Similar to other linguistic competence, pragmatic competence also consists of both the pragmatic knowledge and cognitive processes of L2 learners. With increasing attention on pragmatic performance, in particular, a more comprehensive understanding of the pragmatic competence of L2 learners could be reached if research also focuses on the cognitive processes involved during the pragmatic performance of these learners besides their pragmatic knowledge (e.g., Robinson, 1992; Cohen, 2005; Ren, 2014; Chen, 2015). Such research would yield more information about the reasons underlying language choices and productions related to pragmatic competence (Gass and Mackey, 2000; Li and Gao, 2017).

Currently, one particular focus in L2 pragmatics is understanding pragmatic learning needs in an English for academic purposes (EAP) setting. Studies have investigated the cognitive processes of L2 students that are involved in spoken and written communication in various EAP contexts (e.g., Chen, 2015) and various speech acts, such as apologies, complaints, and requests (Woodfield, 2010, 2012). With the rise of a task-based approach to L2 pragmatic competence (Taguchi and Kim, 2018), pragmatic tasks, such as role-plays, with concrete communicative goals have been considered as viable pragmatic research instruments as they can tap into language use in real-life contexts. Accordingly, the scope of L2 pragmatic competence is well-represented by this model. For this reason, This study investigated the cognitive processes of L2 learners that are involved in role-play-based pragmatic performances in order to reveal these cognitive processes more accurately.

The cognitive processing of an L2 learner is extremely complex and multidimensional (Bi, 2015, 2017). To this end, the study argues that the majority of previous research on the cognitive processes of L2 learners in their speech acts are informed by pragmatic theories rather than the theories of learner strategy research. In order to fully understand the nature of the cognitive processes underlying L2 pragmatic performance, more studies connecting theories in diverse disciplines (e.g., language learning, psychology, and metacognition theories) to pragmatic cognitive processes are necessary.

## Literature Review

### Cognitive Processes From a Pragmatic Perspective

To date, the majority of studies on the pragmatic cognitive processes of learners have primarily relied on L2 pragmatic theories. In addition, most pragmatic researchers (e.g., Robinson, 1992; Cohen and Olshtain, 1993; Ren, 2014; Chen, 2015) used retrospective verbal reports (RVRs) to examine the cognitive processes in pragmatic production. This group of researchers has revealed that a number of mental activities occur in the minds of L2 learners during different speech acts. Notably, the researchers paid close attention to the cognitive processes of L2 learners regarding their sociopragmatic knowledge and pragmalinguistic knowledge production, which characterize two key theoretical dimensions of pragmatic competence (Leech, 1983). For instance, a longitudinal study by Ren (2014) on the variations in the cognitive processes of L2 learners reported that one effective strategy was the development of the control learners have over-attention to pragmatic knowledge through the application of additional cognitive processes that can control and regulate other processes. However, these studies did not present specific findings related to pragmatic cognitive processes. For instance, there were no detailed explanations about what sociopragmatic or pragmalinguistic cognitive processes were employed by learners in order to successfully complete real-life pragmatic tasks in academic contexts.

Furthermore, a closer look at the most recent investigations of cognitive processes in L2 pragmatic competence shows that there seems to be a lack of representation in the L2 pragmatic construct. For instance, Chen (2015) used the RVRs approach to examine the cognitive processes of L2 learners in an email task involving different requests to the lecturers in their university. Fifteen Chinese EFL learners reported a number of politeness, planning, and evaluation processes to compose their emails. The contributions of these studies to the current understanding of cognitive processes in pragmatic performance are evident. However, the instruments used in these previous studies do not fully represent L2 pragmatic competence. L2 pragmatic competence entails complex and multi-faceted components, including abilities to take and organize turns effectively when speakers accomplish pragmatic actions in conversation (Kasper, 2006; Taguchi and Roever, 2017). Accordingly, the multi-faceted dimensions of L2 pragmatic competence need to be reflected in instruments if researchers aim to uncover more accurate and comprehensive cognitive processes involved in L2 pragmatic performance.

A study by Li and Gao (2017) was one of the few that attempted to use interactive data such as simulated role-play tasks to explore the metapragmatic awareness of L2 learners in pragmatic performances. The findings revealed that the learners self-monitored their pragmalinguistic and sociopragmatic knowledge, which led to their metapragmatic awareness. Meanwhile, the self-evaluational behaviors of learners also played a role in managing task demand and intentional linguistic choice to respond to a particular communication setting. The study was one of the few studies that examined both performance data and processing data to illustrate what learners said and why in given situations. Nonetheless, the study only focused on the self-regulation dimension of cognitive processes. Thus, further investigations on both self-regulation, as an umbrella notion, and strategies, as concrete mental processes, are needed.

Another insightful recommendation from Li and Gao (2017) is that more in-depth investigations looking at the cognitive processes of learners before and during their pragmatic performances are needed. This view is consistent with Oxford (2017), who suggested that the cognitive processes of learners in language performance would go through the “forethought, performance, and self-reflection phase” (p. 72). However, little empirical research identified how these cognitive processes occurred differently during each phase of the L2 performances.

Overall, although researchers have started bridging the gap between pragmatic performance and cognitive processes, few human cognition theories were applied in the previous studies to understand the cognitive processes underlying pragmatic performance. Hence, aside from L2 pragmatic theories, cognitive processes in the pragmatic competence of L2 learners should be cross-referenced to other research traditions, such as learner strategy research, metacognition, and human information processing theories.

### Cognitive Processes From a Learner Strategy Perspective

Since 1990, numerous studies have started documenting the cognitive processes employed by L2 learners. In the literature, there are many different terms for the cognitive processes of learners, such as learning/learner strategies, cognitive processes, metacognition, and self-regulation (for a review, see Rose et al., 2018). To avoid confusion, the current study used cognitive processes as the macro category for mental processes. However, the cover term, strategies, included the subset of cognitive processes, for instance, the commonly known language learner strategies (e.g., metacognitive and cognitive strategies) and language-related strategies (e.g., pragmatic strategies). This classification also conformed to how pragmatic researchers referred to mental activities as cognitive processes in their research.

Compared to other language skills, pragmatic strategies have drawn relatively less attention (Oxford, 2017; Cohen, 2020). Nonetheless, researchers (e.g., Cohen, 2005) have already developed the taxonomies of pragmatic strategies. In the taxonomy, Cohen proposed the following types of strategies: cognitive, metacognitive, social or affective strategies, communication, and cover strategies learners use in their speech acts. The taxonomy provided us with a useful guideline to investigate cognitive processes in speech acts. However, the taxonomies were based on general language-use situations rather than specific language-use contexts, such as an academic setting. Therefore, more empirical research is needed in order to validate the taxonomies and develop a more fine-tuned measure for cognitive processes in L2 pragmatics (Cohen, 2005, 2020).

Regarding cognitive processes in specific situations, in the past decade, the pragmatic competence of L2 learners in EAP context has begun to draw attention among some strategy researchers (e.g., Cohen and Sykes, 2013; Youn and Bi, 2019) due to the increasing numbers of international students pursuing a University degree in English-speaking countries. The research reported that many international students may not be adequately prepared for cultural, societal, and interpersonal communications. Cohen and Sykes (2013) recommended that explicit attention to language learner strategies in the field of intercultural pragmatics and intercultural education could enable students to deal with complex real-life situations in their academic life. In their study on the effectiveness of strategy-based instruction, the results suggested that familiarizing learners with strategies would make a difference in their pragmatic performance. The study by Youn and Bi (2019) was one of the few empirical studies that quantitatively investigated how L2 learners at varying levels of pragmatic performance used metacognitive, cognitive, and pragmatic strategies to complete a range of pragmatic tasks in an academic setting.

Despite the scarcity of research, these studies have provided us with some empirical evidence of cognitive processes in L2 pragmatic performances from the learner strategy perspective. In order to have a more accurate understanding of the cognitive processes of L2 learners', empirically validated pragmatic tasks should be adopted as research instruments (Cohen and Sykes, 2013). The current study argue that a pragmatic task, such as a simulated role-play, can be used to examine the cognitive processes of learners during pragmatic interactions.

### Investigating L2 Cognitive Processes Using a Task-Based Approach

In terms of research methods in L2 pragmatics, a discourse completion task (DCT), one of the most popular data elicitation methods, has been predominant. A typical DCT involves a brief situational description and asks participants to respond with a speech act utterance (e.g., apology, refusal) either in a spoken or written format. Since participants are asked to provide a single response within a planning time, they do not directly interact with interlocutors while completing DCTs. Thus, DCTs are practical to administer as researchers can systemically fluctuate contextual variables in the scenarios. For example, Ren (2013) used computer-based DCTs to examine the refusal strategies and cognitive processing of learners. Ren designed eight DCT scenarios with photos and audio conversations to increase the authenticity of the situations. However, the single-turn refusal responses of learners in DCTs do not involve the online pragmatic performance in spoken interaction. Despite the common use, DCTs are not suitable for eliciting a wide range of L2 pragmatic abilities (Golato, 2003; Kasper, 2008; Cohen, 2020).

Alternatively, L2 pragmatic researchers have started paying attention to a task-based approach to designing research instruments that reflect real-life communicative situations and enable learners to negotiate for meaning to elicit a multi-dimensional scope of pragmatic competence (e.g., Taguchi and Kim, 2018). Despite such strengths, pragmatic tasks need to be validated. This means that identified pragmatic tasks need to be meaningful for learners to elicit a full scope of L2 pragmatic competence. To this end, explicit attention to gathering valid evidence of pragmatic tasks used in previous research on pragmatic strategy is still lacking. For example, in her study on the pragmatic cognitive processes of Chinese learners, Chen (2015) used an email request task to a professor, which is known to be meaningful for learners in an academic context. Nonetheless, it is only the request being in a specific written genre (i.e., email) that limits the generalizability of the findings. Ways in which learners manage task-specific communicative demands while interacting with interlocutors to accomplish pragmatic actions remain unknown as well. It is highly possible that learners rely on distinct pragmatic strategies to cope with the cognitively demanding nature of online task-based pragmatic performances.

In order to address these research gaps, the study relied on validated role-play tasks involving several speech acts (e.g., request, refusal, agreement, and disagreement) (Youn, 2015) in this study. Youn gathered a range of validity evidence for role-play tasks, which were designed based on the task-based pragmatic learning needs of EAP stakeholders (e.g., students and teachers) (Youn, 2018). Quantitative and qualitative evidence suggested that the role-play tasks elicited a wide scope of pragmatic competence, ranging from pragmalinguistic knowledge to interactional resources. Taken together, this study argues that the role-play tasks used in this study adequately measured L2 pragmatic competence and are accordingly suitable for the empirical validation of task-based cognitive processes.

Since previous studies have not systematically researched the pragmatic performances and cognitive processes of L2 learners in real-life situations, studies using empirically validated role-play tasks would reveal more meaningful and accurate cognitive processes. Furthermore, no matter how learners apply pragmatic knowledge in real-life situations, this knowledge contains the complex and distinct cognitive processes that depend on when they are used, such as before and during a performance (Li and Gao, 2017). Therefore, cognitive processes need to be examined in terms of the different stages of a pragmatic performance (e.g., cognitive processes before and during the pragmatic performance). To this end, this study answered the following research questions.

1. What metacognitive and cognitive strategies did L2 learners employ to deal with task-based pragmatic performance?
2. What pragmatic strategies did L2 learners employ to deal with task-based pragmatic performance?

## Methods

### Participants

Prior to the data collection, the Institutional Review Board (IRB) in a University in North America had approved the ethical application of this study. In order to comply with the guidelines set down by the IRB, the researchers first announced the research and called for research participants at an English Intensive Program and other degree-seeking programs. Thirty international students with varying first language (L1) backgrounds voluntarily participated in the study. Upon completion, each participant received monetary compensation (20 US$). The selection criteria included the participants being L1s, English proficiency, and the length of living in English-speaking countries. Although the University had a large percentage of students from a similar cultural background (e.g., Chinese), this study aimed to explore the pragmatic performances of students within various cultures. Therefore, the dominance of a specific L1 group was minimized. In the study, the L1s included Chinese (*n* = 11, 37%), Russian (*n* = 4, 13%), Arabic (*n* = 5, 17%), Indonesian (*n* = 4, 13%), Romanian (*n* = 2, 7%), Spanish (*n* = 1, 3%), Portuguese (*n* = 1, 3%), Urdu (*n* = 1, 3%), and Hindi (*n* = 1, 3%) participants.

Given that the role-play tasks in this study represented real-life communication in an academic setting, at least a low-intermediate level of English proficiency was required for all participants. According to the alignment of Test of English as a Foreign Language (TOEFL) Internet-based test (iBT) Scores with the Common European Framework of Reference (CEFR) levels, the TOEFL scores of lower-intermediate level learners are between 57 and 86 and advanced learners are above 110. In this study, the TOEFL iBT scores of the learners ranged from 57 to 112. Of the 30 participants, 14 were graduate-level students; 16 were undergraduate students. For the purpose of this study, the pragmatic performance level was determined based on the rating of two trained raters using the rating criteria from previous studies (Youn, 2015; Youn and Bi, 2019). The pragmatic performances of the participants were rated based on content delivery, appropriate language use, sensitivity to the situation, engaging interaction, and turn organization. Therefore, the criteria were different from other general language proficiency tests. The study identified 17 high-level and 13 low-level learners (see Table 1). However, lower-level learners did not represent true beginner learners.

**Table 1:** Pragmatic performance levels of participants.

**Levels**	**ID (Gender *F/M*)**
High	ID 1 (*F*), ID 3 (*F*), ID 4 (*F*), ID 5 (*F*), ID 6 (*F*), ID 7(*F*), ID 8 (*F*), ID 9 (*M*), ID 10 (*M*), ID 12 (*F*), ID 15 (*F*), ID 16 (*F*), ID 20 (*F*), ID 21 (*F*), ID 23 (*F*), ID 24 (*M*), ID 28 (*F*)
Low	ID 2 (*M*), ID 11 (*M*), ID 13 (*F*), ID 14 (*F*), ID 17 (*M*), ID 18 (*M*), ID 19 (*M*), ID 22 (*F*), ID 25 (*M*), ID 26 (*M*), ID 27 (*M*), ID 29(*F*), ID 30 (*F*)

### Role-Play Tasks

All participants completed three pragmatic role-play tasks (two professor role-plays and one classmate role-play). The role-play tasks reflected real-life situations in an academic context that require pragmatic competence. The validity of the role-play tasks, in terms of their quantitative function and construct representation, was examined (Youn, 2015). Through the large-scale needs analysis on task-based pragmatic learning in an EAP context (Youn, 2018), the most needed and relevant situations that various stakeholders (students and teachers) wanted to learn were identified. The role-play situations involved two interlocutors, professor, and classmate (see Appendix A). For the professor situation, the participants completed two speech acts. First, they requested a recommendation letter with a short due date from a professor in a visit during office hours. Secondly, they refused the request of the professor to change an upcoming class presentation schedule due to a scheduling conflict. As for the classmate role-play, two participants played as classmates who were working on a class project and interacted on how and where they planned to meet for an upcoming project. In order to ensure some degree of authenticity and standardization, role-play cards for each participant were not shared and various contingencies were embedded in the role-play cards. For example, for the refusal role-play with a professor, the participants did not expect a request from a professor. They were given a schedule constriction (i.e., an upcoming final) which most likely led them to refuse the request of the professor.

### Data Collection Procedure

#### Task Completion

Each participant individually met with the professor interlocutor to complete the two role-plays with professors. Two participants were randomly assigned to complete the classmate role-plays, with the proficiency level controlled. The order of role-play tasks was counterbalanced. Each role-play interaction lasted ~2–3 min on average.

#### Retrospective Method for Eliciting Strategic Processing Data

In order to elicit the strategy use of each participant before and during the completion of the tasks, the current study used stimulated-recall to ask learners to report their thinking processes at various stages. In language learner strategy research, quantitative questionnaire analysis has been a dominant approach (Rose et al., 2018). However, if we conducted a preliminary investigation into the mental activities of learners, especially to explore under-researched abilities such as pragmatics, a qualitative approach would provide much more in-depth information (see Woodrow, 2005; Tseng et al., 2006; Rose et al., 2018). In this study, the participants first practiced reporting their strategy use after reading the instructions of each role-play situation. Then, each participant completed the first role-play situation. To minimize the memory effect, each participant was asked to report the strategies right after completing each role-play task. A set of questions was prepared, which was consistently used for all participants. However, for some participants who were noticeably answered the minimum in their responses, more guiding questions (e.g., asking to explain more) were asked. The data were all audio-recorded and transcribed for subsequent analysis.

### Coding Reported Strategy Use

The purpose of the qualitative analysis of the elicited verbal reports data was to identify distinct types of strategies across the participants. For the analysis of the processing data, various steps with a bottom-up approach were taken to identify the distinct cognitive processes.

As a first step, the researchers examined the data to get a general sense of what was happening in the data. Then, the apparent features and themes that emerged from the data were identified. For example, in a response to the question of the researcher to report any strategy utilized during the role-play request performance, one of the participants reported: “*I really want to give a space for the professor to think or decide, not to push. So I kinda try to give options or maybe like if the professor cannot do it's totally okay too*.” The researchers agreed that this response contained an independent thought that represented an explicit strategy. Here, the learner listed explicit social rules (e.g., not to push and giving options for a professor to consider when responding to the request of a student) when talking with a professor. As the researchers went through the rest of the data, the orientation of the participants to appropriate social rules in specific contexts was noticeable, which resulted in a concrete strategy type, situation-related sociopragmatic strategy (see the Results section for details).

While examining the retrospective data of the learners, a list of strategy uses and cognitive processing literature was consulted in establishing a coding scheme for this study. The study referred to investigations in pragmatic strategy use research (e.g., Cohen, 2005; Ishihara and Cohen, 2010) and cognitive processing research in pragmatics (e.g., Robinson, 1992; Cohen and Olshtain, 1993; Ren, 2014; Chen, 2015; Li and Gao, 2017). These strategy taxonomies and existing coding schemes were considered as a prerequisite for comparability with the current study.

Lastly, the codes for distinct strategies that appeared across participants at different performance levels were identified. After multiple rounds of careful revisions, the final coding scheme (see Appendix B for the detailed coding scheme) was developed. The researchers coded half of the data from the verbal reports until accuracy rates over 90% in terms of inter-coder reliability were accomplished. In doing so, a cyclical process of coding, which involved the trial of coding schemes, revision, and recoding the data, was taken to ensure coding accuracy. After finalizing the coding scheme, the researchers independently coded the remaining data. While some undecided coding cases were found, the researchers examined the cases together to reach a final agreement.

## Results

This study examined the cognitive processes of learners underlying task-based pragmatic performance. The analyses mostly focused on the retrospective data of higher-level learners as they were more likely to elicit a wide range of strategies to effectively complete the tasks. The results revealed the emergence of different cognitive processes. Based on the existing literature and verbal report data from the students, the following strategies were identified: cognitive, metacognitive, and pragmatic strategies. Table 2 illustrates the number of participants and their raw frequency of strategy use. Using situation-related sociopragmatic strategies, evaluating performance, and assessing task-related situations were the most frequently reported strategies. It can be noted that high-level learners reported more strategy use in almost all categories. With regard to cognitive strategies, the most common strategy used by higher- and lower-level learners was comprehending, while, unlike, lower-level learners, higher-level leaners tended to link their prior knowledge and put themselves in the task situations. In terms of metacognitive strategies, there was a clear trend that higher-level learners were more strategic, since they were more likely to set plans, evaluate their plans, performances, and emotions, and assess task situations. For the pragmatic strategies, both groups employed a number of situation-related sociopragmatic strategies. However, higher-level learners differed from lower-level learners when utilizing substantially more situation-related pragmalinguistic strategies during task completion.

**Table 2:** Raw frequency of strategy use.

**Categories**	**Strategies**	**High (*n* = 17) frequency (% out of total number of strategies)**	**Low (*n* = 13) frequency (% out of total number of strategies)**	**Total (*n* = 30) frequency**
Cognitive strategies	Comprehending	21 (57%)	16 (43%)	37
	Linking to prior knowledge or experiences	20 (77%)	6 (23%)	26
	Recalling appropriate L2 linguistic knowledge	3 (43%)	4 (57%)	7
	Put yourself in the task situation	11 (85%)	2 (15%)	13
Metacognitive strategies	Setting goals	20 (59%)	14 (41%)	34
	Evaluating performance	38 (59%)	26 (41%)	64
	Evaluating the execution of plans	5 (83%)	1 (17%)	6
	Evaluating emotional state/effect	20 (65%)	11 (35%)	31
	Assessing task-related situations	36 (69%)	16 (31%)	52
Pragmatic awareness and strategies	Pragmatic awareness	17 (63%)	10 (37%)	27
	Situational-related pragmalinguistic strategy	41 (84%)	8 (16%)	49
	Situational-related sociopragmatic strategy	49 (58%)	35 (42%)	84
	Situational-related interactional strategy	11 (73%)	4 (27%)	15

The following section will present transcriptions of the verbal reports illustrating the strategy use and differences in the approach used by higher-level and lower-level learners. These excerpts were chosen for the insight they provide into the cognitive processes of these students in completing various pragmatic tasks.

### Reported Cognitive Strategies of Learners

For the purpose of this study, cognitive strategies referred to conscious mental activities when using language and world knowledge to complete pragmatic tasks. The following types of cognitive strategies were identified in the study.

#### Comprehending

In general, comprehending strategies are commonly used in a wide range of L2 language-use situations, as learners need to identify main ideas and the attitudes of speakers, translate, predict, and make inferences in their language use (Phakiti, 2007). In this study, comprehending strategies often occurred before the learners started the task performance at the pre-task stage, as illustrated below:

*I read the situation introduction and the task for us to complete and then I will see the content of the task like, then I will see what suggestions can I provide to him based on my knowledge or experience*. (ID21 Pre-task)

ID21 tried to comprehend the role-play tasks. She reported that she understood the task situations and gained a full understanding of the requirements quickly. Although the wording of the task requirements was simple, the higher-level students appeared to start making inferences about the communicative situations in the tasks. The data also suggested that the comprehending of learners usually goes along with their pragmatic awareness (see the section on pragmatic strategies). For instance, ID16 indicated that, after understanding the tasks, she started to think about “*how to be courteous and at the same time be in good relationship with a professor*.”

During the role-play tasks, the learners also reported comprehending strategies frequently. For example, when interacting with another participant on a classmate role-play, ID16 tried to understand what an interlocutor meant and then used proper “neutral phrase” to reply, as illustrated below:

*Sometimes I understood what she said but I thought maybe that's not what she really thinks and I should change somehow the flow of the conversation, and maybe not and that's why I tried to use some neutral phrases and then she replied again and then from those phrases I understood that, okay, that's what she means exactly, and I just followed her ideas*. (ID16 Classmate Interaction)

#### Linking to Prior Knowledge or Experiences

This cognitive strategy was prominent among all learners in terms of its occurrence rate and reported use throughout the different stages of their performances. The learners reported that they dealt with task situations based on their personal knowledge or prior experiences. Since the role-play tasks reflected commonly occurring academic language-use situations, many learners tended to refer to their own experience from their lives in university. At the pre-task stage, ID21 reported:

*Yeah because we had some like group projects to do in classes and we had to discuss with our group members about the meeting and yeah I think I had some previous experience like this*. (ID21 Pre-task)

When the learners completed the classmate role-play, which involves the negotiation of a meeting time for a group project, ID9 noted the preference of choosing a comfortable schedule, as seen below:

*But for me personally if I had a set schedule or something, I was going to stick with that schedule because it's what more comfortable for me*. (ID9 Classmate Interaction)

#### Recalling Appropriate L2 Linguistic Knowledge

The learners used this strategy to invoke L2 linguistic knowledge, such as appropriate vocabulary and grammar. Then, the learners analyzed different linguistic choices for the interactions. For instance, ID17 reported that, to be polite and respectful to the professor, he used particular words and grammatical conventions over others. The higher-level learners tended to retrieve their linguistic knowledge and use the knowledge appropriately:

*Yeah to make sure that my sentence is clear that I get vocab and get grammar and I can pronounce the word to make him think it's clear and understand*. (ID17 Professor Interaction)

However, for lower-level learners, they mainly focused on only linguistic knowledge. For instance, ID11 mentioned in the task completion: “*[I thought about] grammar and spelling words because I'm not so good at English, as you can see*.” In contrast, the higher-level learners tended to focus on pragmalinguistics and sociopragmatics (see discussion on pragmatic strategies) rather than just linguistic knowledge when performing pragmatic tasks. This finding is consistent with previous research suggesting that upper-intermediate-level learners think about pragmatics more than linguistic planning (Hassall, 2008).

#### Put Yourself in the Task Situation

This strategy was applied when the learners imagined themselves in a particular situation to solve various real-life communication problems. This strategy appears to be unique to role-play tasks. Compared with other tasks (e.g., DCT), the task-based approach creates a *de facto* real-life situation. Consequently, the learners were more likely to imagine themselves as characters in the performance. The higher-level learners utilized this strategy during the pre-task stage, as illustrated in the following example:

*I was just thinking about myself when I'm really in that situation. So what I would say first since I don't know what the role card will be so I was just thinking the opening and maybe the closing part*. (ID6 Pre-task)

Meanwhile, the learners also employed this strategy throughout their task performances. For instance, when talking to the professor, ID4 reported to put herself in similar academic conversations, which helped her to respond to the professor. She noted the following.

*I'm just understanding the content and then think about the situation, put myself in that kind of situation. If I were in this kind of condition what would I say and again it is easier because you give me your question, I just need to respond. So that's the strategy I guess* (ID4 Professor Interaction)

Furthermore, the learners constantly thought about what they would do if similar situations had occurred. The results repeatedly showed that this strategy can effectively help students to be more capable of coping with academic communications properly, if such a strategy is appropriately employed.

### Reported Metacognitive Strategies of Learners

Metacognitive strategies refer to conscious goal-directed processes and are considered as an executive cognitive function that regulates the thinking and decision-making processes of a person during a pragmatic performance. In this study, the following metacognitive strategies were reported:

#### Setting Goals

This strategy reflected the degree to which the students felt they utilized the higher-order capacity of identifying explicit objectives and goals before or during the task-based pragmatic performance. This strategy was mostly employed at the pre-task stage or at the beginning of the performance. The following excerpt shows that the higher-level learners were likely to set a clear goal to follow or to be guided by certain appropriate pragmatic norms. However, this was mainly observed only in the performances of higher-level learners. For instance, ID5, at the pre-task stage, set task completion goals, which included being respectful, figuring out what needs to be accomplished, and identifying the role-play task expectations as reported in the excerpt below:

*I think I tried to be like, basic standard is try to be respectful to the partner, like the professor or my classmates, and tried to have the real conclusions, like if we want to discuss about the meeting time, we need to make it short and make it an efficient conversation, not like we talk a lot and we don't know when we finally meet*. (ID5 Pre-task)

#### Evaluating

Evaluating strategies refer to the “past and current actions or performance, such as assessing levels of difficulties, self-questioning, evaluating performance/product accuracy” (Phakiti, 2007, p. 3). In this study, the learners reported to evaluate their performances, execution of plans, and emotional status.

##### Evaluating Performance

The higher-level learners continuously evaluated their performances for potential areas for correction. Apart from evaluating their speaking performances in general, the learners tended to evaluate whether or not their performances were appropriate by linking their pragmatic processes (see the following section on pragmatic strategies). As the excerpt below shows, ID15 constantly evaluated her performance throughout the tasks. She expressed concern over whether appropriate social norms were applied in her interaction with the professor. At the same time, she was very conscious about the accuracy of her language. The excerpt demonstrated that higher-level learners know what particular social and linguistic knowledge should be implemented in the performance and what should be avoided.

*I said more questions whether I'm asking questions correctly, if it is a polite way of addressing the professor, and whether the order it's of as it was for some point in previous tasks in the written emails, can I know where your office is, not where is your office. I'm all the time monitoring for these interactions because it's a little bit different from my first language, Russian, so I have to make sure, and especially articles as well. I have to make sure I have articles everywhere. I'm sure I made many mistakes everywhere*. (ID15 Professor Interaction)

##### Evaluating Execution of Plans

Due to the highly cognitive demanding nature of the role-play tasks, the learners evaluated whether their plans went as planned. For instance, apart from evaluating the role-play performance itself, ID15 reported that she constantly thought about the initial goals to make sure that what she had to say and how she said it was done properly. As seen below, ID15 set the goal of interacting with the professor appropriately. In order to successfully execute this plan, she needed to stay on track to find ways to help the professor to complete the role-play.:

*Before I think the goal was achieved as well because professor ask me whether I have time and according to the case I didn't have time. And also trying to be polite kind of politely say no. so it was fine with the prof as well. And um so at the end we were trying to find an option and a chance how to, I was trying to find a way to help the professor to deal with the situation*. (ID15 Professor Interaction)

##### Evaluating Emotional State/Affect

The learners evaluated emotional state/affect during the pragmatic performance. For instance, ID15 consciously noticed herself feeling tense in the performance as shown below:

*I was a bit nervous. That's how I feel all the time with talking to the professor, I'm all the time concerned with making a mistake and trying to monitor like output, so and its kind of all information have to be polite, provide exact information when trying to what exactly I want to tell the prof. and it's all this information, maybe that's why I'm a bit nervous*. (ID15 Professor Interaction)

This excerpt shows that the L2 learners were engaged in self-evaluating while still performing the pragmatic tasks. Interestingly, some very strategic learners showed the ability to ease nervousness by quickly shifting their focus back to the tasks. For instance, when interacting with classmates, ID15 evaluated the situation and calmed herself down: “I was feeling much more comfortable with my classmate because I didn't feel this kind of barrier between the professor and the student” (Classmate Interaction). After knowing that the role-play involved two close friends, ID5 again evaluated the emotional status at the moment: “*So I knew that Phoenix is my best friend and I have nothing to be afraid of and in this way I was really relaxed, I was thinking clearly, I didn't have any fear inside*” (Classmate Interaction).

#### Assessing Task-Related Situations

The last type of metacognitive strategy found in this study was assessing task-related situations, which acts as a higher-order executive function. The excerpt below indicates that the learners often evaluated the complexities involved in refusing a request from a professor.

*Rejecting the request from professor was harder. I asked to write… Is it the same scenario? Okay. I asked your help. And then you ask me to do something and I couldn't you know be reciprocal. So I feel like really sorry*. (ID7 Professor Interaction)

Since the role-play tasks elicit task-based pragmatic performance with spoken interaction, the learners monitored the responses of the interlocutors and assessed their expectations. As seen in the excerpt above, ID7 assessed the difficulty of rejecting the request of a professor and elaborated why it was not expected to reject it, especially after the professor agreed to write a recommendation letter. It is worth noting that this strategy was not previously identified in any theoretical taxonomies or empirical research. This study suggests that higher-level L2 learners assess task-specific interactional contexts to ensure context-appropriate speech act performance.

### Reported Pragmatic Awareness and Strategies of Learners

Pragmatic cognitive processes refer to conscious mental processes involved in task-based pragmatic performances, including the general awareness and online processes of learners. The four types of pragmatic cognitive processes found in this study were closely related to various components of pragmatic knowledge.

#### Pragmatic Awareness

The learners reported that pragmatic awareness was related to either the target language or the individual culture of the learners, which occurred during the performance rather than at the pre-task stage. For example, ID5 (higher-level) reported the language use involved in his interaction with his professor as “keep it somewhat formal, but informal at the same time” by explaining his current relationship with a professor as a graduate student in the culture of the target language, which is different from his previous relationship in his own culture, as seen below.

*So mainly it's more professional kind of environment when you use the first name. And with my professors… and you have open hours when you come in and you talk to the professor most of the time, you really become friends… I tried to* (ID5 Professor Interaction)

ID10 displayed pragmatic awareness during the classmate interaction focusing on the different degrees of formality in the US compared his L1 culture.

*In my mind, I think at this point after being in the US an English speaking country for more than a year I was not having too much to strategize right now. But I remember when I just came to the US I had similar situation. So the culture back home is like totally different. And people are more informal. That's what I feel*. (ID10 Classmate Interaction)

Other learners also reported thought processes related to their own culture and linguistic repertoire in L1, which contrast with L2 English.

*In my own language, in Indonesian language there are steps of words. So this word is higher in position than this one although the meaning is the same. So if I want to talk to the professor or something, I use the higher word.”* (ID9 Professor Interaction)

*In Taiwan we don't even call the last name. We just call, “hi teacher, hi professor.” And then also how to use this polite form… we use “please.” It already sounded like you're polite. But in English it's also you have to use a lot of hedging*. (ID7 Classmate Interaction)

ID9 was oriented to the polite words in her own L1, which led her to realize polite words in English. With regard to address terms, ID7 reported a difference between her own culture and the target culture, displaying an explicit awareness of cross-linguistic differences. Although the amount of information provided is different depending on the levels of the learners', the majority of learners, regardless of their levels, displayed pragmatic awareness.

#### Situation-Related Pragmalinguistic Strategy

The learners were oriented to two important dimensions of pragmatics, pragmalinguistics, and sociopragmatics (Leech, 1983). Pragmalinguistics concerns the linguistic means necessary to accomplish pragmatic meaning and comprehend the utterances of speakers. Similar to pragmatic awareness, the learners reported pragmalinguistic strategies during the performances and not at the pre-task stage. As seen in the excerpts below, the learners actively referred to various linguistic repertoires, such as formulaic expressions and modal verbs. ID9 noted “*trying to be more polite. … mostly primarily through the intonation when talking to a professor*.” At the same time, they shifted the formality when talking with the classmate by using “*Direct [language]*,” such as “*Okay what about this? Okay I like that* (ID9).” With regard to linguistic resources specific to speech acts, the higher-level learners reported different resources used when refusing the request of a professor. For example, ID7 provided very explicit linguistic resources as seen in the following.

*I didn't think I could tell my professor in the face. …I'd always still say, “I'll check” and indeed I will check and they maybe email my professor and say, “I'm sorry that they said to be on time” and then just kind of “can you please help me.” But…I won't say, “I won't do it, I don't think so*.” (ID7 Professor Interaction)

ID7 specified numerous linguistic means, such as an elaborated account and indirect expressions using modal verbs. These higher-level learners were very strategic in terms of choosing appropriate linguistic resources in given contexts. Although this study primarily focused on higher-level learners, it is worthy to note that there was a noticeable absence of pragmalinguistic strategies among lower-level learners.

#### Situation-Related Sociopragmatic Strategy

Parallel to pragmalinguistics, the learners were also oriented to the sociopragmatic. Sociopragmatics focuses on the understanding of social rules and contextual variables that influence language use and interpretation (Leech, 1983). In other words, one needs to understand social and contextual variables, such as a relationship between speakers and the context in which situations occur, to be pragmatically appropriate. It should be noted that the sociopragmatic strategies reported were somewhat different from the pragmatic awareness strategies discussed above. The sociopragmatic strategies contain the orientation of the learners to specific contextual variables, such as the degrees of imposition and the different relationships between interlocutors. Interestingly, compared to other pragmatic strategies, the learners were oriented to the sociopragmatic dimension even before completing the tasks. The excerpt below shows that ID6 noticed the different degrees of formality for each role-play and explained the informal nature of classmate role-play because the conversation was between friends.

*I think this will come more naturally than the previous one because this one is more formal so this one says informal so I didn't really think much. This is just with my friend*. (ID6 Pre-task).

Right after the performance, the learners actively reported the sociopragmatic strategies they utilized during their performances. ID10 expressed an explicit awareness of the fluctuation of the manner of speaking depending on the relationship with an interlocutor.

*I think it's a lot different because when talking to the classmate I was kind of assertive and I said “okay, this is what I want” and I might want your opinion on it, but maybe I'm not able to change my opinion. But with a professor I was choosing which word say that I'm okay with anything that you're doing*. (ID10 Professor Interaction)

Unlike the pragmalinguistic resources, the lower-level learners also displayed sociopragmatic strategies. ID13 (low-level) reported “*because you are a teacher I sort of keep polite*.” Although this reported sentence was noticeably shorter compared to those of higher-level learners, it still included a reference to the relationship between a speaker and a hearer (i.e., teacher), which is an important situational variable in the reason for being polite in given situations.

#### Situation-Related Interactional Strategy

Finally, situation-related interactional strategies were identified, which primarily characterize the explicit strategy of learners in dealing with interactional demands in conversation. It should be noted that no one mentioned this strategy before the performance or during the pre-task stage. All identified interactional strategies were reported right after the performance. Because the targeted performances involved interacting with various interlocutors, the learners managed the conversational demands consciously. For example, as seen below, some learners were oriented to the appropriate order of sequencing the turns. ID5 listed how he intentionally placed the greeting sequence (“*asking how the week was*”) at the beginning of the conversation with a professor, which displays his knowledge of turn-taking rules in conversation.

*So informal would be I'm using the first name, I'm asking how the week was, was it busy or not. That would give me a better understanding if the professor can accomplish the ask that I want him to do. So I asked at the very beginning. If the professor would tell me he's busy, I would change my way of deliverance, of my inquiry from the very beginning*. (ID5 Professor Interaction)

ID7 also noted that she displayed hesitation when refusing the request of a professor using distinct prosodic properties. ID7 displayed an explicit knowledge of turn-taking and noted “*if you sa*y, “*oh that would really be great if you can do it*” *and I would say*, “*okay, I would try, I would try*.” *So I already feel that that part of me already prepared*.” This reported strategy indicates that ID7 possessed an explicit knowledge of providing an appropriate answer to a previous turn (i.e., turn-taking).

## Discussion And Limitations

A wide range of cognitive processes was identified in the study, including processes, discussed in existing taxonomies and empirical studies, and other processes uniquely reported before and during the task-based performances. Regarding the cognitive processes from the learner strategy perspective, according to Cohen and Sykes (2013), little research on the strategies of learners in L2 pragmatic performances is available. The present study provided an empirical basis for the taxonomy by Cohen (2005) of pragmatic strategy and what L2 pragmatic competence entails. For instance, in the study, the learners employed cognitive strategies to assist themselves in retrieving linguistic knowledge and experiences to complete the pragmatic tasks. At the same time, they utilized metacognitive strategies to monitor and evaluate their pragmatic knowledge application. The learner strategy literature in other language skills have suggested that metacognitive strategies are higher-level processes that regulate cognitive strategies (e.g., Phakiti, 2007; Bi, 2015, 2017, 2020). The current study also found similar patterns in pragmatic performances. Furthermore, it is interesting to note that the metacognitive and cognitive strategies occurred throughout the task performances, that is, before and during the performance. This provides further evidence that pragmatic competence is not only composed of knowledge, but cognitive processes play a vital role in this competence.

The study has also revealed additional cognitive processes that are rarely discussed in previous research and theories. For instance, cognitively, the learners tended to put themselves into the task situations; metacognitively, they were more likely to assess the task-related situations. These strategies may not be exclusively reported in our study, but they are certainly key strategies for coping with role-play pragmatic tasks. Role-play tasks are more cognitively demanding compared to other pragmatic instruments (e.g., DCTs) and involve resolving real-life communicative situations, which, in turn, evoke a wide range of L2 pragmatic knowledge (Kasper, 2008). Accordingly, it can be argued that the learners are in need of utilizing additional strategies for task-based pragmatic performances.

The pragmatic strategies reported in the study illuminated the cognitive processes underlying pragmatic performance and the nature of L2 pragmatic competence. The pragmatic awareness of learners reflected the recognition of appropriate sociocultural and linguistic norms both in their own culture(s) and target-language culture. Pragmatic awareness stored in the long-term memory of learners potentially contributes to the regulation of online situation-related pragmalinguistic and sociopragmatic strategies in a concerted effort to perform L2 speech acts. Between the pragmalinguistic and sociopragmatic dimensions, the learners, regardless of their levels, utilized sociopragmatic strategies, which were also attended both before and after the pragmatic performance. On the other hand, pragmalinguistic strategies were more commonly reported among the higher-level learners. In addition, with regard to the interactive nature of role-play performance, the findings revealed that the high-level learners actively utilized the interaction-related strategies. This means that the learners were aware of how to utilize interactional repertoire (e.g., how to start the conversation, how to sequence context-relevant information, and how to respond to a question) in a context-fitting manner for a successful pragmatic performance. This finding supports the current expanded view of L2 pragmatics-in-interaction, which includes the ability to jointly accomplish pragmatic actions contingent upon an unfolding course of conversation (Kasper, 2006; Taguchi and Roever, 2017).

Another contribution of the current study is that, through a comprehensive investigation from the perspectives of both learner strategy and pragmatic research, the findings have provided further empirical evidence for the exceptionally complex and interrelated nature of the cognitive processes of learners. The results highlighted the role of L2 metacognitive strategies for pragmatic awareness. Li and Gao (2017) emphasized the role of self-monitoring and self-evaluative behaviors on the pragmatic awareness of learners. This study further endorsed the view. For instance, we found that the higher-order processes, such as metacognitive and pragmatic awareness, taking a concerted effort would significantly impact the pragmatic choices of L2 learners. Nevertheless, we also argued that learner strategies coexist with pragmatic awareness and situation-related pragmalinguistic, sociopragmatic, and interactional strategies.

The limitations of this research must be acknowledged. First of all, since it was an exploratory study, the results lacked generalizability. Secondly, the reported cognitive processes were based on the retrospective verbal report. Although the retrospective data were collected immediately after the performance data, the reported strategies were still delayed. Consequently, they may not reflect the strategies the students actually used. Future research may use think-aloud methods to ask participants to verbalize their thoughts while performing a pragmatic task. Such methods can provide rich data when investigating the mental processes underlying complex pragmatic task performances. Third, the performance data of the learners themselves were not discussed in relation to the reported cognitive processes due to limited space. Connecting cognitive processes with pragmatic performances would be necessary for further investigations. Follow-up studies that address these issues will advance the research on cognitive processes.

## Pedagogical Implications

Despite the above limitations, the findings of this study can strengthen pedagogical practices regarding the value of strategy instruction in L2 pragmatic learning. Cohen (2019) pointed out that, although pragmatic rules related to the target language culture have been taught in English as a foreign language (EFL)/English as a second language (ESL) classes, students may still not know “when, why, and how to use them” (p. 141). Unlike other language skills, pragmatic instruction and learner strategies for successful pragmatic performance have not been explicitly included in L2 classrooms (Taguchi and Roever, 2017; Youn, 2018). However, L2 learners can benefit greatly from learning different types of strategy (Cohen, 2019).

The current study provided further empirical evidence to support the notion that successful learners tend to employ a variety of metacognitive and cognitive strategies (see Table 2). Additionally, higher-level learners utilized more situation-related pragmalinguistic strategies to accurately and appropriately deliver their messages, which were not commonly employed by lower-level learners. This adds much-needed evidence to support the “widely debated relationship between strategy use and language learning success” (Rose et al., 2018, p. 157). Consequently, when teaching L2 pragmatics, teachers may introduce effective metacognitive strategies to learners to regulate and control their performance. Also, learners can be taught to employ cognitive strategies such as referring to past cultural and linguistic experiences to perform L2 pragmatics appropriately.

Our study also found that pragmatic strategies assisted L2 learners in understanding the expected target sociocultural norms and interactional demands to accomplish pragmatic actions in spoken interaction. For instance, in section Reported Pragmatic Awareness and Strategies of Learners, learners reported pragmatic awareness in their own culture and target-language culture and thus chose appropriate linguistic expressions and placed turns appropriately to achieve shared understanding and maintain the continuity of the interaction. Given that the learners only reported the interaction-related strategies after the performance, it is possible that the learners might not be consciously aware of such strategies until they are engaged in the interaction. In addition, lower-level learners rarely reported interactional strategies. which emphasizes the need for explicit strategy instruction. Accordingly, teachers need to help raise and sharpen the L2 pragmatic awareness of learners. Especially, since learners have the awareness of their first languages and target-language cultures, specific strategy instructions are useful to help learners shift from their own culture to the target culture. Students should also be trained to retrieve appropriate sociopragmatic, pragmalinguistic, and interactional strategies for successful pragmatic performances in various academic settings.

## Data Availability Statement

The raw data supporting the conclusions of this article will be made available by the authors, without undue reservation.

## Ethics Statement

The study was reviewed and approved by Human Research Protection Program. The participants provided their written informed consent to participate in this study.

## Author Contributions

The author confirms being the sole contributor of this work and has approved it for publication.

## Conflict of Interest

The author declares that the research was conducted in the absence of any commercial or financial relationships that could be construed as a potential conflict of interest.

## Publisher's Note

All claims expressed in this article are solely those of the authors and do not necessarily represent those of their affiliated organizations, or those of the publisher, the editors and the reviewers. Any product that may be evaluated in this article, or claim that may be made by its manufacturer, is not guaranteed or endorsed by the publisher.
