# Supplementary material for: Cognitive Processes of ESL Learners in Pragmatic Role-Play Tasks in Academic Settings
Source: Front Psychol. 2021 Aug 30;12:586588. doi: 10.3389/fpsyg.2021.586588 (PMC8436886; doi:10.3389/fpsyg.2021.586588)

**Appendix A: Example role-play tasks and cards**

**Professor role-play**

**Situation:** You have an appointment with a professor Morgan Brown today to ask for a recommendation letter for a scholarship for international students from your department and to ask a few questions about a course project. Your professor is meeting with you outside of the office hour since you have a class during the office hour. Now you’re about to visit your professor. You just enter to a professor’s room.

**Task**: You will receive role-play cards that describe what you’re going to tell your professor. Please have a conversation with your professor naturally.

**For undergraduate participants**: This professor teaches Economy 101 that you’re taking this semester.

**For graduate participants**: This professor is one of the faculty members in your department. Although he/she is not your advisor, you’ve known this professor for about 1 year and you’re currently taking a course from this professor.

**Role-play Card (Meeting time)**

| **Jessie** |  | **Professor** |
| --- | --- | --- |
| 1. After greeting, **ask for a recommendation letter** for the department scholarship that you will apply. The letter is due in one week |  |  |
| **Jessie** |  | **Professor** |
|  |  | Respond to the request. Inform the student that you will write a letter and ask when the due date is, if the student doesn’t tell you.  Inform students that you have a conference next week and you’re leaving tonight. Tell him/her that you will do your best to submit the letter by the deadline, but ask the student if the letter can be submitted a bit late. |
| **Jessie** |  | **Professor** |
| 1. Respond to what the professor says and **tell the professor that you will check with your department office.** |  |  |
| **Jessie** |  | **Professor** |
| 1. Inform the professor of two options of providing a letter, **hard copy or electronic submissions** through a website. **Ask for the professor’s preference.** |  | Prefer an electronic submission as you will be traveling. |

**Classmate role-play**

**Situation**: After class, you’re going to talk with your classmate who is doing a class project (article presentation) regarding **when your group members will meet** to discuss the project. The third member (Tom) is absent today in class. Your presentation is next Friday.

**Task**: You will receive role-play cards that describe what you’re going to tell your classmate. Please have a conversation with your classmate naturally.

**Role-play Card (Meeting time)**

| **Jessie** |  | **Phoenix** |
| --- | --- | --- |
|  |  | 1. As approaching to Jessie, start a conversation about an upcoming class project (article presentation).   **Suggest** discussing an appropriate meeting time. Propose one available time slot based on your schedule. |
| **Jessie** |  | **Phoenix** |
| 1. **Look at your schedule**, Respond to a Phoenix’ question. |  |  |
| **Jessie** |  | **Phoenix** |
|  |  | 1. Respond to Jessie’s time availability **based on your own schedule.** |
| **Jessie** |  | **Phoenix** |
| 1. You need to leave soon since you have another class soon. So, whether you found a good time or not, **suggest** asking the third member (Tom)’s opinion to make a final decision. |  |  |
| **Jessie** |  | **Phoenix** |
| 1. Respond what Phoenix says |  | 3. Respond what Jessie says |

**Appendix B**: Coding schemes for pragmatic strategies (Youn & Bi, 2019)

| **Categories** | **Strategies** | **Operational Definition** |
| --- | --- | --- |
| Cognitive strategies:  Conscious mental activities involved in using language and knowledge to deal with pragmatic tasks | Comprehending task | Comprehending task instructions to aid or improve performance |
|  | Translation | Translating target language into first language |
|  | Linking prior knowledge or experience | Utilizing and linking from prior knowledge or experience |
|  | Recalling appropriate L2 linguistic knowledge | Recalling general linguistic knowledge that is not directly related to L2 pragmatics |
|  | Putting yourself in the task situation | Relating yourself in relation to task situations |
| Metacognitive strategies:  Conscious goal-directed processes | Setting goals | Setting overall goals to aid or improve successful performance |
|  | Planning | Planning specific procedures necessary for successful performance |
|  | Evaluating their own performances | Evaluating their own task-based performance |
|  | Evaluating the execution of plans | Evaluating how plans functioned during performance |
|  | Evaluating emotional status | Evaluating emotional status during performance |
|  | Assessing task-related situations | Evaluating difficulties and complexities involved in task situations |
| Pragmatic strategies:  A wide range of strategies related to L2 pragmatic knowledge required in tasks | Pragmatic awareness: Target-language culture | General pragmatic awareness based on the knowledge on target-language culture |
|  | Pragmatic awareness: One’s own culture | General pragmatic awareness based on one’s own culture |
|  | Situation-related sociopragmatic strategies | Strategies related to contextual variables and cultural norms in various social contexts (i.e., sociopragmatics) specific to pragmatic tasks situations either planning or during performance |
|  | Situation-related pragmalinguistic strategies | Strategies related to various conventions of language use (i.e., pragmalinguistics) specific to pragmatic task situations either planning or during performance |
|  | Sitution-related interactional strategies | Strategies related to accomplishing successful social interaction with interlocutors either planning or during performance |


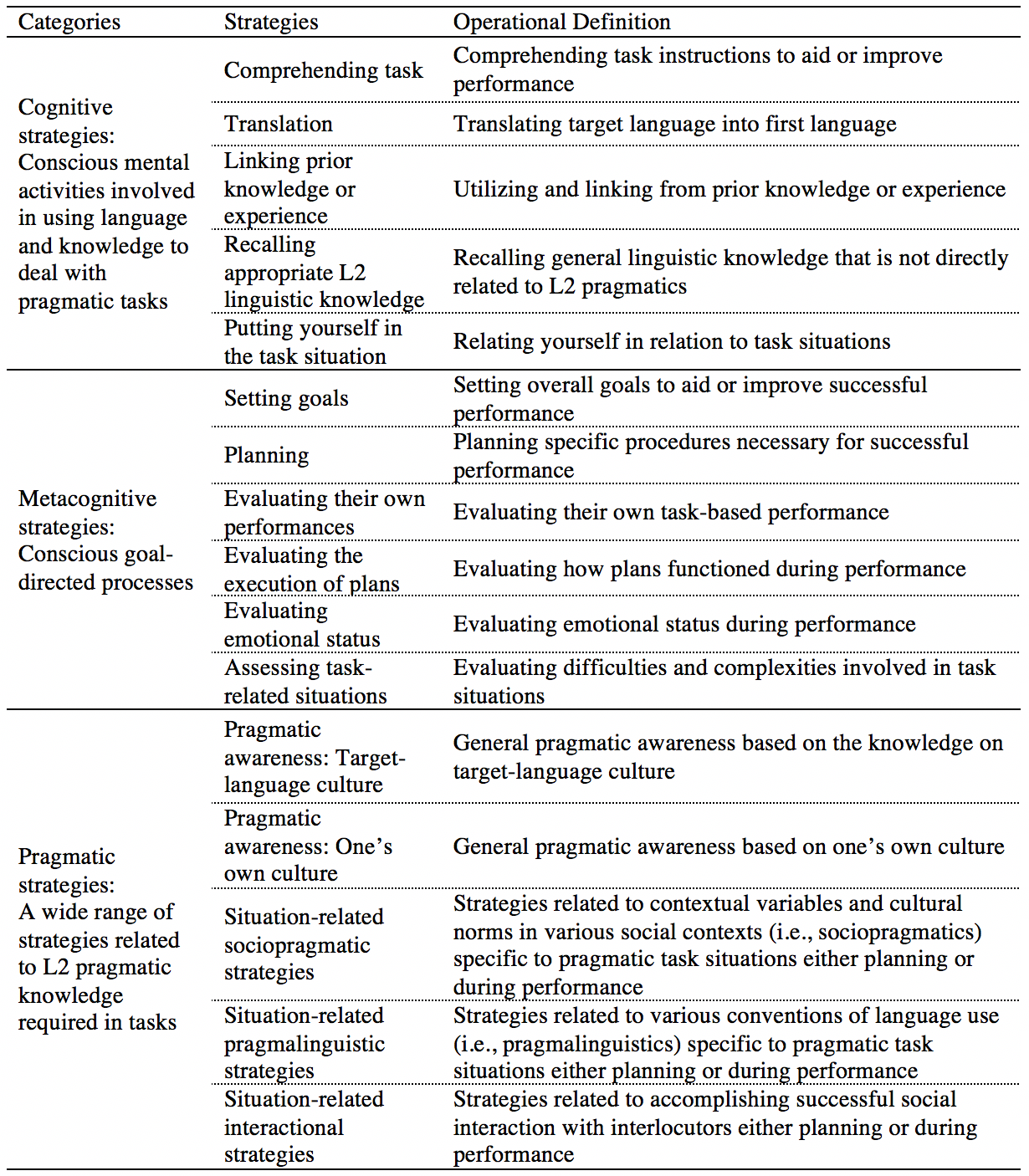

Supplement: Supplementary file 1 [file Data_Sheet_1.docx]
